# Correlation of scar localization between cardiac magnetic resonance imaging and electro-anatomic mapping at electrophysiology study in patients with cardiac rhythm management devices

**DOI:** 10.1186/1532-429X-17-S1-P244

**Published:** 2015-02-03

**Authors:** Rajeev R Fernando, Nisreen F Ali, Lara Bakhos, Jeffrey R Winterfield, David Wilber, Osamah Abdallah, Mark Rabbat, Mushabbar A Syed

**Affiliations:** Department of internal medicine, Loyola University Medical Center, Maywood, IL USA; Department of Cardiology, Loyola University Medical Center, Maywood, IL USA

## Background

Cardiac magnetic resonance imaging (CMR) can accurately delineate myocardial scar for substrate guided ventricular tachycardia (VT) ablation. Susceptibility artifact from cardiac rhythm management device (CRMD) generator and leads can significantly affect image quality limiting the evaluation of myocardial scar and foci of VT. We conducted a retrospective study to correlate scars from CMR to the voltage mapping obtained at electrophysiology study (EPS).

## Methods

We studied 27 patients with CRMD who underwent CMR and EPS at Loyola University Medical Center between 11/2012 to 9/2014. Baseline demographics and clinical parameters are outlined (Table 1). A total of 432 left ventricular (LV) segments were analyzed for wall motion and late gadolinium enhancement using the AHA 17 segment model excluding the apical cap. The presence of right ventricular (RV) scar and wall motion abnormalities was also recorded. Data from the EPS included the type of access (endocardial and/or epicardial), the presence and location of scar, and the VT foci.

**Table 1:** Baseline clinical parameters

Age (years)	58.52 +/- 14.82
Male/Female	(25/2)
Ischemic cardiomyopathy	16(59%)
Non-ischemic cardiomyopathy	11 (41%)
Diabetes mellitus	4(15%)
Hypertension	14(52%)
Hyperlipidemia	14(52%)
Smoking	10(37%)
Body mass index (kg/m2)	26.5+/- 6.3
Body surface area (m2)	2 +/-0.14
Left Ventricular Ejection Fraction (% +/- standard deviation)	41.23+/-16.73
Left ventricular end-diastolic volume index (% +/- standard deviation)	133.45 +/-14.2
Right Ventricular Ejection Fraction (% +/- standard deviation)	44.31 +/- 12.06
Right ventricular end-diastolic volume index (% +/- standard deviation)	96.84 +/- 35.89
Pacemaker/ICD	2/25

## Results

Of the 432 LV segments, 127 (29.3%) were affected by artifact precluding assessment for myocardial scar. The mid anterior (81.4%) and basal inferior (3.7%) segments were the most and least affected by artifact respectively. CMR scar parameters were compared with EPS scar mapping (Table 2). CMR was able to identify LV scar, particularly endocardial scar, in most cases despite the presence of device-related artifact. In 17/22 patients (77.3%) with CMR identified LV scar, the LV endocardial scar location on CMR was comparable to the voltage map at EPS. In only one patient was LV endocardial scar missed on CMR in a non-evaluable segment due to artifact, although that segment did have a wall motion abnormality. Exclusion of LV endocardial scar by CMR is associated with a low diagnostic yield of endocardial mapping at EPS. In 18/22 (81.8%) patients with inducible VT, the focus of VT arose from an area delineated as scar on CMR. In the remaining 4 patients, only one patient had inducible VT unrelated to MRI scar. Of the ten RV scars identified at EPS, only 4 were detected by DE-MRI.

**Table 2:** Comparison of MRI and EPS parameters

CMR parameter	EPS parameter	Co-efficient of correlation	P-value	95% confidence interval for r	Sample size
CMR-All LV scar	EPS-All LV scar	0.59	p<0.0025	0.24-0.8	24
CMR-LV endocardial scar	EPS-LV endocardial scar	0.83	p<0.0001	0.64-0.92	24
CMR-LV epicardial scar	EPS-LV epicardial scar	0.65	p=0.078	-0.09-0.93	8
CMR-LV wall motion abnormality	EPS-LV endocardial scar	0.49	p=0.0092	0.14-0.73	27
CMR-RV scar	EPS-RV scar	0.57	p=0.0031	0.22-0.79	24
CMR-RV wall motion abnormality	EPS-RV scar	0.42	p=0.028	0.05-0.69	27

## Conclusions

CMR can be a valuable tool in localizing LV scar prior to EPS for ablation of VT. However, 29.3% of LV segments are not evaluable due to artifact from device generator (anterior wall in 65.4%). Regional wall motion abnormalities in non-evaluable left and right ventricular segments can guide identification of scar at EPS.

## Funding

None.

